# Development of a Strain-Specific Real-Time PCR Assay for Enumeration of a Probiotic *Lactobacillus reuteri* in Chicken Feed and Intestine

**DOI:** 10.1371/journal.pone.0090208

**Published:** 2014-02-27

**Authors:** Verity Ann Sattler, Michaela Mohnl, Viviana Klose

**Affiliations:** 1 University of Natural Resources and Applied Life Sciences, Department for Agrobiotechnology, Tulln, Austria; 2 BIOMIN Research Center, Tulln, Austria; Catalan Institute for Water Research (ICRA), Spain

## Abstract

A strain-specific real-time PCR assay was developed for quantification of a probiotic *Lactobacillus reuteri* (DSM 16350) in poultry feed and intestine. The specific primers were designed based on a genomic sequence of the strain derived from suppression subtractive hybridization with the type strain *L. reuteri* DSM 20016. Specificity was tested using a set of non-target strains from several sources. Applicability of the real-time PCR assay was evaluated in a controlled broiler feeding trial by using standard curves specific for feed and intestinal matrices. The amount of the probiotic *L. reuteri* was determined in feed from three feeding phases and in intestinal samples of the jejunum, ileum, and caecum of three, 14, and 39 day old birds. *L. reuteri* DSM 16350 cells were enumerated in all feeds supplemented with the probiotic close to the inclusion rate of 7.0×10^3^ cfu/g, however, were not detected in *L. reuteri* DSM 16350 free feed. In three day old birds *L. reuteri* DSM 16350 was only detected in intestinal samples from probiotic fed animals ranging from 8.2±7.8×10^5^ cfu/g in the jejunum, 1.0±1.1×10^7^ cfu/g in the ileum, and 2.5±5.7×10^5^ cfu/g in the caecum. Similar results were obtained for intestinal samples of older birds (14 and 39 days). With increasing age of the animals the amount of *L. reuteri* signals in the control animals, however, also increased, indicating the appearance of highly similar bacterial genomes in the gut microbiota. The *L. reuteri* DSM 16350 qPCR assay could be used in future for feeding trials to assure the accurate inclusion of the supplement to the feed and to monitor it's uptake into the GIT of young chicken.

## Introduction

Lactobacilli are widely used as probiotics in animals [1]. For application as feed additive in poultry production, a probiotic bacterium is commonly isolated from the intestine of healthy chickens and further selected for specific beneficial properties [2]. Identification of the probiotic is important to discriminate it from related strains with different properties. Differentiation can be achieved with molecular typing methods such as pulsed-field gel electrophoresis (PFGE) [3] or random amplified polymorphic DNA (RAPD)-PCR [4]. However, these techniques rely on isolation and cultivation capabilities of organisms and are of limited use in complex microenvironments such as feed or the gastro-intestinal tract (GIT). For evaluating the efficacy and persistence of a probiotic strain, it is important to assure correct inclusion rates in the feed and to trace the introduced strain through the GIT. Methods to monitor the probiotic should be strain-specific, quantitative and applicable for analysis of feed and GIT samples. Quantitative PCR (qPCR) is a technique that has been used to detect several bacterial species in food [5], rumen [6] or faeces [7]. It's high sensitivity enables quantification of microorganisms with low abundance within an environmental sample. The challenge in qPCR development is designing primers that specifically target species or strains of interest, despite the presence of closely related bacteria. Efficiency and accuracy of the qPCR depend on DNA quality. The main obstacles for good quality DNA are co-extraction of PCR-inhibitory substances from the environmental matrix and inefficient recovery of total genomic DNA from the bacterial community [8]. To create qPCR standard curves for absolute quantification of microorganisms, environmental samples are often spiked with a known amount of target cells before DNA is extracted. This allows a more precise determination of microorganisms of interest in a complex sample. Animal feed contain a diverse bacterial population originating from soil, water, or dust where the feed plant was grown, processed and/or stored [9]. *Enterobacteriacae* were mainly found in commercial poultry diets [10], while lactobacilli proliferate best under moist and anaerobic conditions and are predominantly present in grass silage feed [11]. Thus, lactobacilli are not expected to be prevalent in rather dry mashed or pelleted poultry feed, which makes it easier to specifically detect these, when added to grain feed. In contrast, lactobacilli are a prominent group of the autochthonous microbial community prevalent in the upper GIT part of chicken [12], [13]. The genus *Lactobacillus* is taxonomically very complex and known for its extreme phenotypic and ecological diversity [14]. To differentiate closely related *Lactobacillus* strains based on 16S rRNA gene sequence is difficult due to high sequence homology in variable regions of the gene [15]. As an alternative to this gene, several reports describe the development of strain-specific primers using RAPD [16], [17], [18], [19], [20] or by identifying phage-related sequences [21]. Suppression subtractive hybridization (SSH) is a method to identify genomic DNA fragments that are present in one but not another closely related strain [22], [23], [24]. This method is especially useful, when genome sequence information is lacking for the strain of interest. In combination with qPCR, these unique genomic markers might be used to track probiotics from the feed through the animal's GIT. So far, only few studies have reported the use of a genome-hybridization based method combined with qPCR for identification and quantification of probiotics *in vivo* [25], [26]. This study describes the development of a strain-specific qPCR assay for detection of the chicken derived strain *Lactobacillus reuteri* LR (DSM 16350), which was isolated and evaluated as a probiotic strain within the European Union project “C-EX” (QLK-CT-2002-71662) for the use as feed additive in young chicken [2]. To our knowledge, SSH in combination with qPCR was used for the first time for specific quantification of a *Lactobacillus* strain in environmental samples. Primers were tested for specificity with a set of non-target *L. reuteri* strains. Applicability of the qPCR assay was evaluated in a feeding trial with broiler chickens by using standard curves, specific for feed and intestinal matrix. Presence and amount of the probiotic were determined in feed from three different feeding phases, and additionally the probiotic was monitored in three compartments of the GIT (jejunum, ileum, caecum) of three, 14, and 39 day old birds.

## Materials and Methods

### Bacterial strains and growth conditions

Suppression subtraction hybridization (SSH) was performed with the probiotic strain *Lactobacillus reuteri* (DSM 16350) as tester, which is mentioned hereafter by the code LR, and with the type strain *Lactobacillus reuteri* (DSM 20016) as the driver. Other *L. reuteri* strains used for specificity testing were of distinct sources, either purchased from strain collections or previously isolated from animal intestinal samples (Table 1). All strains were grown in de Man Rogosa Sharpe medium (MRS; Oxoid, Hampshire, UK) under semi-anaerobic conditions at 37°C for 24 h. Electro competent cells *Escherichia coli* ElectroMAX DH10B (Invitrogen, Carlsbad, CA, USA), used for cloning, were grown at 37°C on Luria Bertani agar (LB; Oxoid, Hampshire, UK) supplemented with ampicillin 100 mg/ml.

**Table 1:** *L. reuteri* (LR) qPCR specificity test (C_T_ values and melting curve analysis) using non-target strains from various sources, purchased from culture collections and isolated from intestinal samples.

*L. reuteri strains*	Source of origin	C_T_ mean	Tm (°C)	MC properties +/−
DSMZ^a^ 16350^b^(LR)	chicken intestine	10	78.7	+
DSMZ 8533	lab strain [41]	>31	b.t.	−
DSMZ 12246	Chr. Hansen strain	>31	78.4*	−
DSMZ 17509	rat gut	>36	78.9*	−
LMG^c^ 18238	chicken	>40	b.t.	−
DSMZ 20015	manure	>40	81.8	−
DSMZ 20016^T^	human intestine	n.d.	-	−
DSMZ 20053	human faeces	>40	b.t.	−
DSMZ 20056	rat faeces	n.a.	-	−
LMG 22879	laying hen, cloacae	>31	b.t.	−
CA2	pig intestine	>36	78.9*	−
LRS	pig intestine	>36	78.8	+
F2	pig intestine	>36	79.1	−
R8A	pig intestine	>31	78.6*	−
R20	pig intestine	>31	80.5	−
R22	pig intestine	>36	80.6	−
R31	pig intestine	n.a.	-	−
R36	pig intestine	>31	79.5*	−
S2A	pig intestine	>31	79.1*	−
S4A	pig intestine	>40	80.0	−
S6A	pig intestine	>36	80.8	−
S8A	pig intestine	>36	83.5	−
S11A	pig intestine	>31	80.1	−
S14A	pig intestine	>40	b.t.	−
S21A	pig intestine	>31	b.t.	−
S21C	pig intestine	>31	79.7	−
S24B	pig intestine	>31	76.2	−

C_T_ cycle threshold.

Tm melting temperature.

MC melting curve.

a DSMZ - Deutsche Sammlung von Mikroorganismen und Zellkultur.

b target strain and SSH tester strain.

c LMG – Belgian coordinated collections of microorganisms.

T type strain and SSH driver strain.

n.a. no amplification.

b.t. below threshold (33%).

“-“ MC was either below threshold, showed the formation of one or more products, or showed a shift in Tm>1°C compared to Tm of LR (78.7°C).

“+” MC properties were identical to LR.

*melting curves not reproducible.

### Ethics statement

Feeding trial protocol and animal experiments were approved by the local authority for agriculture ‘Amt der Niederösterreichischen Landesregierung für Agrarrecht’ in accordance with the Austrian act on animal experimentation (1988, BGBL 501/1989). Newly hatched broiler chickens (Ross) of mixed sex were kept in an environmentally controlled poultry house at the Centre for Animal Nutrition (Mank, Austria).

### Feeding trial and sample collection

Newly hatched broiler chickens (Ross) were randomly allocated to eight pens per group with 20 birds per pen (in total 320 birds). Birds from the control group received a standard formulated broiler feed without supplements and birds from the probiotic group received the standard feed supplemented with *Lactobacillus reuteri* LR with a final concentration of 7.0 x 10^3^ cfu/g feed. Birds were fed manually once a day with diet and water available *ad libitum*. Starter feed was given from day 0–14, followed by grower diet from day 15–28, and ending with the finisher feed from day 29–41. Intestinal samples were taken from animals (n = 8 per group and day) at day three, 14 and 39 of the trial, which was equivalent to the age of birds. Contents of jejunum, ileum and caeca were collected in sterile tubes and immediately frozen.

### DNA extraction

DNA from bacterial cultures was extracted following a protocol for Gram- positive bacteria [27]. Cells were lysed with lysozyme (2.5 mg/ml) and Proteinase K (250 µg/ml), cell suspension purified with phenol∶chloroform∶iso-amylalcohol (25∶24∶1), and DNA precipitated in two volumes of ethanol at −20°C for at least 2 h. The DNA pellet was washed with 70% ethanol and dissolved in 50 µl nuclease-free water.

Genomic DNA from about 250 mg intestinal digesta was extracted using the QIAamp DNA Stool Mini Kit (Qiagen GmbH, Hilden, Germany) according to the manufacturer's instructions for pathogen detection. Prior to the kit protocol, samples were incubated with lysozyme (50 mg/ml) for 45 min at 37°C and then homogenized for 10 s at 6000 rpm using Precellys® SK38 bead beating tubes and the Precellys® 24-Dual homogenizer (Peqlab Biotechnology GmbH, Erlangen, Germany).

Microbial DNA was extracted from 20 g feed sample of every feeding phase. To wash off bacterial cells from feed particles, the feed was mixed with 100 ml peptone water containing 1% Triton X-100 and shaken in a flask for 30 min. The mixture was then smashed and filtered through a stomacher bag. Bacterial cells of the filtrate were pelleted and further used for DNA extraction following the protocol for intestinal samples. Isolated DNA was visualized by agarose gel electrophoresis and concentration was determined by NanoDrop ND-1000 spectrophotometer (Peqlab Biotechnology GmbH, Erlangen, Germany).

### Construction of suppression subtractive hybridization (SSH) clone library

For SSH, the PCR-select bacterial genome subtraction kit (Clonetech Laboratories, Mountain View, CA, USA) was used to subtract unique genomic DNA of the probiotic strain *L. reuteri* LR (tester DNA) from the type strain *L. reuteri* 20016^T^ (driver DNA) following the manufacturer's protocol with modifications. The first hybridization step was performed at 55°C for 90 min and the second hybridization at 55°C for 16 h. Primary and secondary (nested) PCR were conducted using the Advantage 2 Polymerase Mix (Clonetech Laboratories, Mountain View, CA, USA), 10 mM dNTP mix, 10 µM of each primer, and 1 µl template DNA. Subtracted PCR products were cloned into pJet1.2/blunt vectors using the CloneJet PCR Cloning Kit (Fermentas, Burlington, CA, USA) and transformed into ElectroMAX DH10B cells by electroporation for 5 sec at 1.8 kV using the GenePulser (Bio-Rad Laboratories, Hercules, CA, USA). Transformants were recovered in liquid LB medium and grown over night on LB agar plates with 100 µg/ml ampicillin. From the SSH clone library, 57 clones were picked and a rapid plasmid preparation was performed as previously described [28]. Briefly, each colony was picked with a tooth pick and suspended in 20 µl 0.2 M NaOH. RNA was removed using RNase (10 µg/μl) by incubation for 7 min at 37°C. The plasmid extract was neutralized by adding 40 µl of 0.1 M HCl. Plasmid inserts were amplified by PCR with primers flanking the insertion site of the cloning vector. Size of the PCR products were analyzed by agarose gel electrophoresis.

### Differential screening of strain-specific SSH products

For differential screening of subtracted PCR products from the SSH clone library, a DNA dot blot hybridization was performed. To generate single stranded DNA (ssDNA), all DNA or PCR samples were melted at 95°C and immediately chilled on ice. One microliter of ssPCR product was spotted onto a neutral BioBond nylon membrane (Sigma-Aldrich, St. Louis, MO, USA) and fixed by UV cross-linker (Stratagene, La Jolla, CA, USA) using the auto-crosslinking mode (1200 mJ×100/cm^2^). Spotted membranes were hybridized with either SSH tester (*L. reuteri* LR) or driver (*L. reuteri* 20016^T^) DNA. Before hybridization, genomic tester and driver DNA were digested with RsaI and then labelled with biotin using the Biotin Decalable DNA labelling Kit (Fermentas, Burlington, CA, USA). Membranes were pre-hybridized with salmon sperm DNA to block unspecific binding sites. Then the membranes were incubated over night at 60°C in hybridization buffer (5×Denhardt's solution, 5 X SSPE buffer, 1% SDS) with 100 ng/ml labelled ssDNA under moderate shaking. To remove unbound and unspecific tester or driver DNA, membranes were washed with a non-stringent buffer (2×SSC, 0.1% SDS) and a stringent (0.1 X SSC, 0.1% SDS) buffer. DNA dots were visualized using the Biotin Chromogenic Detection Kit (Fermentas, Burlington, CA, USA) following the manufacturer's instructions.

### Strain-specific primer design and qPCR assay

Potential strain-specific SSH PCR products were sequenced and checked for sequence similarity by the BLAST web tool of the National Center of Biotechnology Information (NCBI) [29]. Sequences were used for primer design with the Primer Premier 5 software (Premier Biosoft, Palo Alto, CA, USA). A set of primers was tested for specificity and efficiency in an annealing temperature gradient-qPCR with genomic tester DNA and genomic driver DNA as targets. The following conditions were applied: initial denaturation at 95°C for 5 min followed by 45 cycles at 95°C for 15 sec, annealing at 55°C–65°C for 15 sec, and elongation at 72°C for 20 min. To assure that the correct PCR product was amplified, a melting curve analysis was added at the end of the PCR program using the default settings of the realplex^2^ Mastercycler ep-gradient S instrument (Eppendorf, Hamburg, Germany). Amplification was carried out in a 15 µl final volume containing 1 × Mesa Green qPCR MasterMix Plus for SYBR (Eurogentec S.A., Seraing, Belgium), 300 nM of each primer, and 3 µl target DNA. The best performing primer set was chosen for further specificity testing with several *L. reuteri* non-target strains (Table 1) in the qPCR assay at optimal annealing temperature (results not shown).

### Standard curves for LR quantification in environmental samples

For quantification of *L. reuteri* LR in feed and intestinal samples two matrix based standard curves were created. Therefore, *L. reuteri* LR free broiler feed was spiked with 8.0×10^8^ cfu/g and gut digesta with 8.5×10^6^ cfu/g of lyophilized LR cells. DNA was extracted according to the matrix dependent DNA extraction protocol and was serially diluted in nuclease-free water. Standard DNA was amplified by qPCR in triplicates applying the same conditions as for primer specificity testing. Standard curves were generated by plotting cycle threshold values (C_T_) versus equivalent log cell numbers. The amplification efficiencies for feed and gut samples, determined by the slope of the standard curves, were calculated based on the equation *E* = (10^−1/slope^−1) x 100. To test accuracy and application of the qPCR assays *in vivo*, feed and gut lumen samples were collected and analyzed from a feeding trial with broiler chickens as described above. The number of *L. reuteri* LR cells was assessed in triplicates by qPCR and expressed as cfu/g.

## Results

### Differential screening and sequencing of SSH subtracted products

Subtracted PCR products from clone 4 and 19 of the SSH clone library were chosen as potential *L. reuteri* LR specific DNA markers as they showed intense colour after hybridization with labelled tester DNA, but not with labelled driver DNA (Figure S1). Insert sequences of clone 4 (accession KJ152779) and 19 (accession KJ152780) had no significant sequence similarity to any known gene in a BLAST search in the NCBI nucleotide database. The highest similarity hit for clone 4 sequence was given with *L. salivarius* CECT 5713 plasmid pHN1 (47% query cover, E = 3e−43, 81% identity) and for clone 19 sequence with *L. reuteri* I5007 plasmid pLRI04 (15% query cover, E = 9e−47, 93% identity).

### *L. reuteri* LR specific primer

Primers that were designed based on sequences of DNA inserts in clone 4 and 19 were tested for efficiency and specificity using tester and driver DNA in a temperature gradient qPCR assay. The primer pair for clone 4, 21f (5′-CAGGATCGGTAATTGATG-3′) and 190r (5′-TGGATATGGAAGTTCGTC-3′), was specific for LR. The best PCR efficiency of this primer pair was at 56°C annealing temperature, because with this temperature highest fluorescent signal occurred at an early cycle threshold (C_T_) of 10. Specificity was tested under the same conditions and showed that all non-targets (n = 26) had C_T_ values above 31 compared to the specific signal at C_T_ 10. Seven strains had C_T_ values above 36, and five strains C_T_ values above 40. The SSH driver strain *L. reuteri* 20016 and two isolates, one from rat (DSM 20056) and one from pig (R31) showed no PCR amplification product (Table 1). Cycle difference between target LR and non-target strains was at least 21, which corresponds to a cell number of about log 6 per gram sample. Unspecific PCR product formation could be detected for non-target strains by melting curve analysis. Melting temperatures of unspecific products were, however, higher (up to 83.5°C) or lower (down to 76.2°C) compared to the LR specific product melting temperature (78.7°C). Six non-target strains revealed melting curves below threshold (b.t.) and several other strains showed formation of two or more products, displayed by multiple peaks (Figure S2). For some samples melting curves were not reproducible between duplicates (Table 1). The pig isolate LRS was the only strain that could not be distinguished by melting curve analysis from the LR curve, however, by its C_T_ value of 31, compared to the LR C_T_ value of 10. Primers for clone 19 were not as specific as the clone 4 primer pair 21f/190r. The non-target strain LRS was efficiently amplified with clone 19 primers and the melting curve of the resulting product could not be distinguished from the LR melting curve. For this reason, the clone 19 primers were rejected for further analysis.

### Standard curves and limit of detection for environmental samples

**Figure 1:**
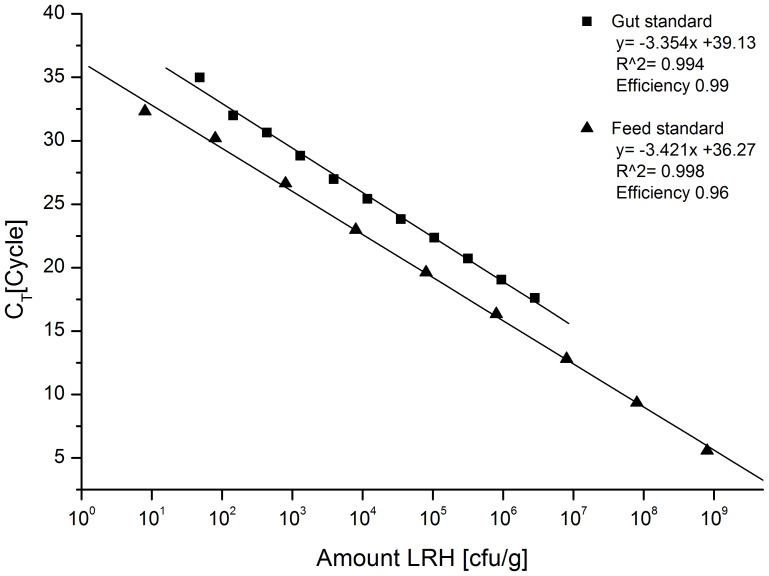
qPCR standard curve for quantification of *L. reuteri* (LR) in feed and gut samples. The standard curves were generated by amplification of serially diluted DNA from feed spiked with 8.0×10^8^ cfu/g and from gut lumen content spiked with 8.5×10^6^ cfu/g.

qPCR standard curves for LR strain-specific quantification in feed ranged from 8.0×10^8^ cfu/g–8 cfu/g, whereas those for LR quantification in gut lumen samples ranged from 8.5×10^6^ cfu/g–48 cfu/g. Amplification efficiency *E* was 96% for feed samples and 99% for gut samples. Based on qPCR results with non-target strains, the limit for specific and reliable detection of LR in any sample was set at C_T_ 31, although the standard curves showed a linear quantification range up to a cycle number of 32 (Figure 1). The detection limit at C_T_ 31 corresponded to 3.5×10^1^ cfu/g, for feed samples, and to 4×10^2^ cfu/g, for gut digesta samples.

### *In vivo* evaluation of the *L. reuteri* LR-specific qPCR assay

The strain-specific qPCR was evaluated with samples from an *in vivo* feeding trial, where LR cell numbers were quantified in feed from three different feeding phases and monitored in gut lumen samples of three days, 14 day and 39 day old control and LR supplemented chicken (LR 7.0×10^3^ cfu/g feed).

#### Feed samples

The starter feed contained 5.4±0.3×10^3^ cfu/g *L. reuteri* LR, the grower feed 2.0±0.1×10^3^ cfu/g *L. reuteri* LR, and the finisher feed 0.8±1.1×10^3^ cfu/g *L. reuteri* LR. Control feeds from each phase were below the detection limit of 3.5×10^1^ cfu/g *L. reuteri* LR.

#### Gut samples (day 3)

*L. reuteri* LR was detected in intestines of three day old birds of the probiotic group, however, not in the control group (Table 2). The average amount of LR in the probiotic group was 8.2±7.8×10^5^ cfu/g in the jejunum, 1.0±1.1×10^7^ cfu/g in the ileum, and 2.5±5.7×10^5^ cfu/g in the caecum. Enumeration of *L. reuteri* LR was possible in each animal and intestinal location (n = 8) from probiotic fed animals. In control animals, the LR cell numbers were below the detection limit in every sample.

**Table 2:** Mean numbers of *L. reuteri* LR in chicken gut samples from animals fed without (control) or with LR (probiotic) over 39 days as determined by strain-specific qPCR.

	*Jejunum*		*Ileum*		*Caecum*	
	Control	Probiotic	Control	Probiotic	Control	Probiotic
**n**	0/8	8/8	0/8	8/8	0/8	8/8
**Day 3**	n.d.	8.2±7.8×10^5^	n.d.	1.0±1.1×10^7^	n.d.	2.5±5.7×10^5^
**n**	3/8	6/8	5/8	8/8	7/8	8/8
**Day 14**	1.2±1.9×10^4^	3.7±9.0×10^5^	1.5±2.8×10^4^	1.5±3.9×10^6^	1.5±2.0×10^4^	3.5±9.0×10^6^
**n**	7/8	8/8	8/8	8/8	6/8	5/8
**Day 39**	2.3±3.2×10^5^	2.0±1.9×10^5^	1.6±2.5×10^6^	4.4±4.5×10^5^	0.6±1.0×10^4^	1.4±2.3×10^4^

n.d. not detected above detection limit for gut sample 4×10^2^ cfu/g.

n number of animals with cell counts above detection limit from a total number of eight animals.

#### Gut samples (day 14)

In 14 day old birds, the average amount of LR in the probiotic group was 3.7±9.0×10^5^ cfu/g in the jejunum, 1.5±3.9×10^6^ cfu/g in the ileum, and 3.5±9.0×10^6^ cfu/g in the caecum (Table 2). LR could be detected in all intestinal samples from the probiotic group except in two jejunum samples. LR cell numbers were 1–2 logs higher in the probiotic group compared to the control group, where the average amount of putative LR detected was similar in all three intestinal locations with 1.5×10^4^ cfu/g. The number of samples with LR signals above detection limit in the control group was 3 out of 8 in the jejunum, 5 out of 8 in the ileum, and 7 out of 8 in the caecum.

#### Gut samples (day 39)

In 39 day old birds, the average amount of LR in the probiotic group was 2.0±1.9×10^5^ cfu/g in the jejunum, 4.4±4.5×10^5^ cfu/g in the ileum, and 1.4±2.3×10^4^ cfu/g in the caecum (Table 2). Quantification of putative LR in control animals showed similar high results compared to probiotic fed animals, with an average LR cell count of 2.3±3.2×10^5^ cfu/g in the jejunum, 1.6±2.5×10^6^ cfu/g in the ileum, and 0.6±1.0×10^4^ cfu/g in the caecum. In the probiotic feeding group, LR could be detected in all samples except in three caecum samples. In the control group, LR signals were also detected in nearly every sample except in one jejunum and two caecum samples.

## Discussion

Guidelines of the Food and Agriculture Organization and the World Health Organization for the evaluation of probiotics in food report that strain identification is important to link the claimed health effect to the probiotic and to enable correct surveillance during efficacy studies [30]. This emphasizes the need for strain-specific identification assays. In this study, DNA sequences that are unique for the genome of the probiotic *L. reuteri* LR were identified with suppression subtractive hybridization (SSH). Two out of 57 SSH clones harboured genomic sequences that were likely specific for LR and that could be used for primer design. Specificity testing with non-target strains showed that only one primer pair (21f/190r) was LR specific. A limitation of the SSH method is the occurrence of a certain portion of false positives [24]. A method called mirror orientated selection (MOS) has been reported to reduce the number of false positive clones from the SSH library [31] and should be considered for future SSH applications. The diversity of different *L. reuteri* strains in the gut is unknown. As it is impossible to isolate and screen all environmental strains, we chose to use a broad array of *L. reuteri* strains of distinct sources for primer specificity testing. Melting curve properties of non-targets were different from those of the LR strain, except for one pig isolate, probably because of homologous primer binding sites in its genome. Melting curves below threshold or with multiple peaks found for several non-target strains are likely due to primer binding to unspecific, partially complementary sequences resulting in inefficient amplification. The cycle threshold (C_T_) difference between LR and non-target samples was equivalent to at least 6 logs of cell numbers. Thus, signals of unspecific targets in environmental samples were considered not to compromise the specific enumeration of *L. reuteri* LR. In order to avoid quantification of false-positive signals, the detection limit was not set according to the linear range of the standard curves but at the lowest cycle threshold that was detected for non-target strains. Detection limits reported in other studies for strain-specific real-time PCR assays varied; e.g. 10^4^ cfu/U for spiked rumen feed and fluid [26] and 10^5^ copies/g for spiked human faeces [20], [32]. In these studies the standard curves were created by inoculating the environmental sample with decreasing concentrations of the target bacterium. This was different from our approach in which the environmental sample was first spiked and then DNA was diluted. This may have led to a relatively low detection limit allowing LR quantification at concentrations below 10^3^ cfu/g, which may be of interest, when the strain is applied as a multi-strain probiotic, where single strains are mixed together [33].

Accuracy and applicability of the qPCR assay were examined in a controlled feeding trial, in which broiler chickens were administered feed supplemented with the probiotic *L. reuteri* LR and control feed. The LR cell number in probiotic-supplemented feeds was close to the theoretical inclusion rate (7.0×10^3^ cfu/g), whereas that in the control feed was below the detection limit. This confirmed that accurate enumeration of LR in feed can be achieved. qPCR analysis revealed that the indigenous feed flora and potentially co-extracted PCR inhibiting substances did not interfere within the assay. In contrast to feed, the species *L. reuteri* is a common member of the gut microbiota in chicken [34]. In three day old birds, enumeration of LR was possible in each bird and intestinal location, indicating that the probiotic was taken up and did spread along the entire GIT. In young chickens, the highest LR cell count was obtained in the ileum. At the age of 14 days, the LR numbers were similar to those from three-day old birds. However, LR signals at a level of 10^4^ cfu/g were also detected in control birds which indicated that two weeks after hatching the gut microbiota of the chickens harboured bacteria with a similar sequence in their genomes. Contamination of analysed control birds was excluded after selective culturing of the LR strain by the use of strain typing methods (data not shown). Microbial colonization of the GI tract begins shortly after hatching [35], [36], and the gut microbiota rapidly develops and becomes more complex as chickens age [34], [37]. When diversity and abundance of microorganisms in the gut increase, at the same time the possibility of unspecific PCR amplification also raises. This is also indicated by the results of 39 day old birds, where the amount of LR found in the control animals was similar high as in the probiotic fed animals. To develop more specific primers, more isolates needed to be screened. However, this approach is limited, as isolation of all *L. reuteri* strains from complex intestinal samples is impossible. Some lactobacilli strains have been genetically labelled e.g. by introducing a fluorescent marker gene [38], [39], or a silent mutation into a chromosomal gene [40]. A genetic label in the bacterial genome provides a way to monitor the specific strain in various environments, but introducing foreign DNA is generally a scientific approach not applicable for feeding farm animals, where this strain would then be released into the free environment. Aside from that, probiotics need to be alive to exert all their beneficial effects in the GIT. In this regard detecting live but not dead cells is of interest and may be achieved by pre-treating the intestinal content with propidium monoacid (PMA), a DNA intercalating chemical that inhibits PCR amplification of non-viable cells [19].

In conclusion, we were able to develop strain-specific qPCR primers using SSH to detect and enumerate *L. reuteri* LR in poultry feed and in the gut lumen of chickens in early days of life. It could be confirmed that qPCR is a sensitive and rapid tool to determine low abundant bacterial strains in samples with mixed microbial population. This technique replaces other genotypic identification techniques that include time consuming isolation of the target strain using selective culturing. In future, the LR specific qPCR assay might be used for feeding trials in order to assure the accurate inclusion of the supplement to the feed and to monitor it's uptake into the GIT of chicken.

## Supporting Information

Figure S1 Dot blot hybridization of SSH clone inserts with A) driver DNA of *L. reuteri* 20016, and B) tester DNA of the target strain *L. reuteri* LR.
(TIF)

Figure S2 Representative melting curves from LR-qPCR specificity test with non-target *L. reuteri* strains showing the formation of unspecific PCR products.
(TIF)
