# Supplementary figures and images for: Development of a Strain-Specific Real-Time PCR Assay for Enumeration of a Probiotic Lactobacillus reuteri in Chicken Feed and Intestine
Source: PLoS One. 2014 Feb 27;9(2):e90208. doi: 10.1371/journal.pone.0090208 (PMC3937354; doi:10.1371/journal.pone.0090208)

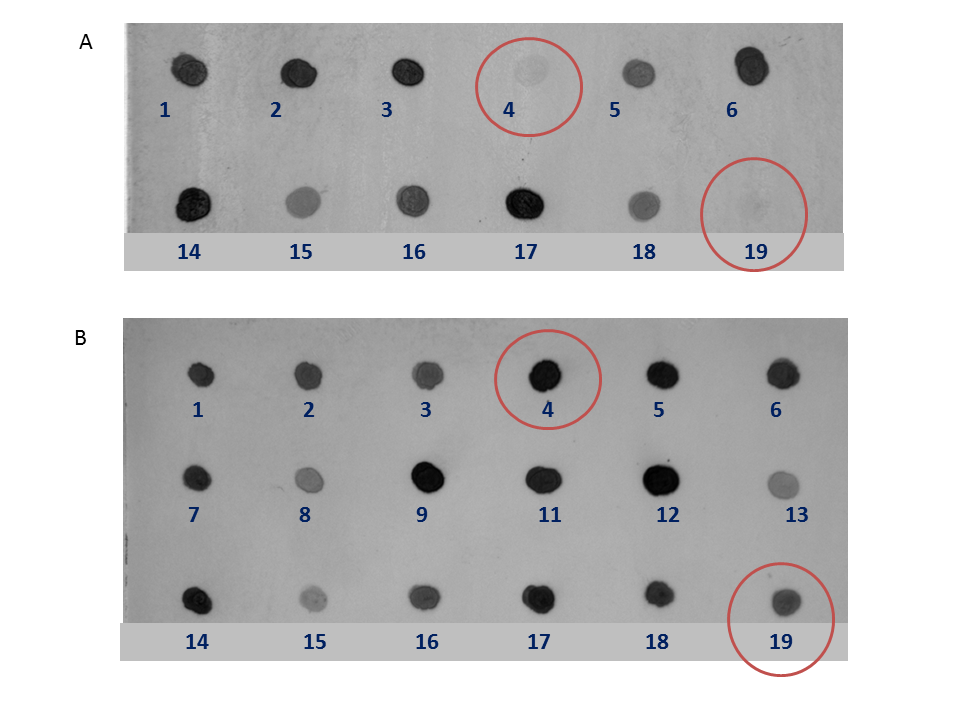

Supplement: Figure S1 — Dot blot hybridization of SSH clone inserts with A) driver DNA of L. reuteri 20016, and B) tester DNA of the target strain L. reuteri LR. (TIF) [file pone.0090208.s001.tif]

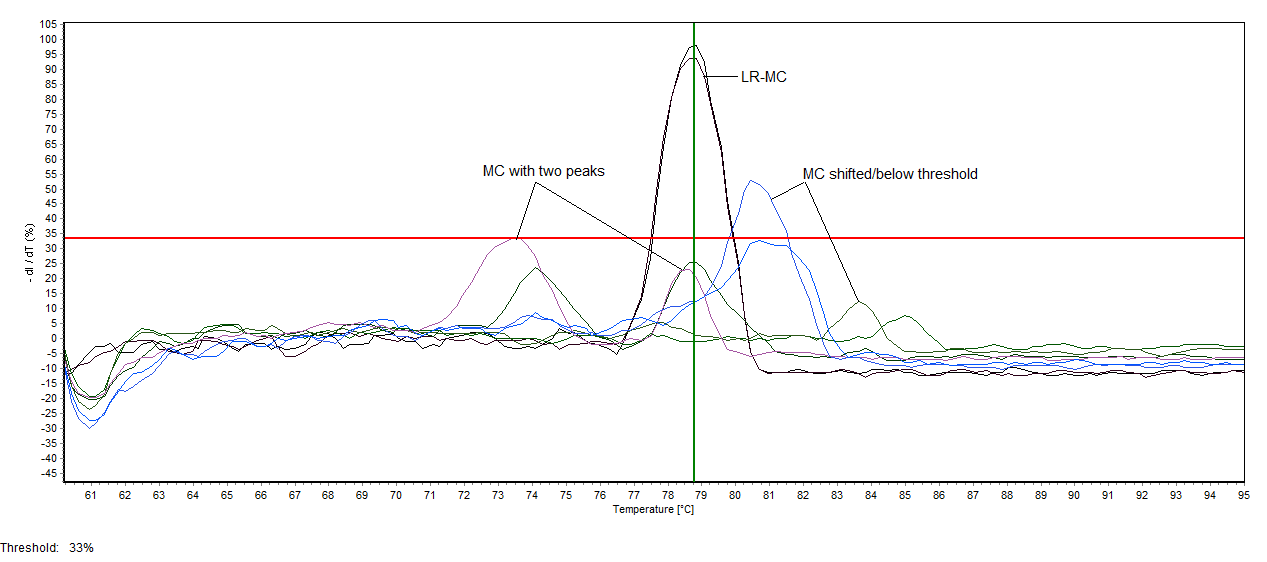

Supplement: Figure S2 — Representative melting curves from LR-qPCR specificity test with non-target L. reuteri strains showing the formation of unspecific PCR products. (TIF) [file pone.0090208.s002.tif]
